# Homology modeling of mosquito cytochrome P450 enzymes involved in pyrethroid metabolism: insights into differences in substrate selectivity

**DOI:** 10.1186/1756-0500-4-321

**Published:** 2011-09-06

**Authors:** Panida Lertkiatmongkol, Ekachai Jenwitheesuk, Pornpimol Rongnoparut

**Affiliations:** 1 Department of Biochemistry, Faculty of Science, Mahidol University, Phayatai, Bangkok 10400, Thailand; 2 Genome Institute, National Science and Technology Development Agency, Thailand Science Park, Pathumthani 12120, Thailand

## Abstract

**Background:**

Cytochrome P450 enzymes (P450s) have been implicated in insecticide resistance. *Anopheles minumus *mosquito P450 isoforms CYP6AA3 and CYP6P7 are capable of metabolizing pyrethroid insecticides, however CYP6P8 lacks activity against this class of compounds.

**Findings:**

Homology models of the three *An. minimus *P450 enzymes were constructed using the multiple template alignment method. The predicted enzyme model structures were compared and used for molecular docking with insecticides and compared with results of *in vitro *enzymatic assays. The three model structures comprise common P450 folds but differences in geometry of their active-site cavities and substrate access channels are prominent. The CYP6AA3 model has a large active site allowing it to accommodate multiple conformations of pyrethroids. The predicted CYP6P7 active site is more constrained and less accessible to binding of pyrethroids. Moreover the predicted hydrophobic interface in the active-site cavities of CYP6AA3 and CYP6P7 may contribute to their substrate selectivity. The absence of CYP6P8 activity toward pyrethroids appears to be due to its small substrate access channel and the presence of R114 and R216 that may prevent access of pyrethroids to the enzyme heme center.

**Conclusions:**

Differences in active site topologies among CYPAA3, CYP6P7, and CYP6P8 enzymes may impact substrate binding and selectivity. Information obtained using homology models has the potential to enhance the understanding of pyrethroid metabolism and detoxification mediated by P450 enzymes.

## Findings

Insecticide resistance is a growing problem in the control of mosquito species that serve as vectors in the spread of malaria. One of the major classes of insecticide detoxification enzymes is the heme-containing cytochrome P450 monooxygenases (P450s). These enzymes are responsible for the metabolism of endogenous and exogenous compounds and the expression of several P450s is increased in insecticide resistant insects [1]. P450 enzymes are thought to promote resistance due to their ability to metabolize insecticidal compounds [2-5] however, the link between increased expression of P450s and insecticide resistance has not been clearly established. Structural information on insect P450s together with investigation of their function in insecticide metabolism may help to increase the understanding of their roles in insecticide detoxification and resistance. To date, crystal structures of insect P450s have not been resolved and structural studies relying on *in silico *homology modeling approaches have been used to gain insight into the molecular basis of insecticide binding [2,6,7].

We previously observed elevated expression of P450 isoforms *CYP6AA3*, *CYP6P7*, and *CYP6P8 *in a laboratory-selected deltamethrin-resistant *An. minimus *mosquito, a major malaria vector in Thailand, relative to the parent susceptible strain [8]. The increase in CYP6AA3 and CYP6P7 transcripts correlated with increased deltamethrin resistance, however CYP6P8 did not enhance resistance to deltamethrin. Our results are consistent with the known overlapping metabolic profiles of CYP6AA3 and CYP6P7 against type I and II pyrethroids [5]. The homology between CYP6P7 to CYP6P8 (61% amino acid identity) does not support a role for CYP6P8 in pyrethroid metabolism [5]. In this study, homology modeling of *An. minimus *CYP6AA3, CYP6P7, and CYP6P8 enzymes was conducted and molecular docking was performed with various insecticide compounds. Our analysis was able to predict the molecular basis of P450 activity against pyrethroid compounds and has the potential to assist in the investigation of new compounds that can bypass resistance due to P450 enzymes.

## Methods

Amino acid sequences of CYP6AA3 (GenBank: AAN05727.1), CYP6P7 (GenBank: AAR88141.1), and CYP6P8 (GenBank: AAR88142.1) were aligned against protein structures deposited in Brookhaven Protein Data Bank (PDB) [9] using PSI-BLAST. Crystal structures of ligand-free CYP3A4 (PDB: 1TQN) [10], CYP2C8 (PDB: 1PQ2) [11], and CYP2C9 (PDB: 1OG2) [12] were used as templates since their sequences were most similar to the target P450s (14-33% primary sequence identity). The templates structures do not contain residues in N-terminal membrane-binding domain and thus the first 25 residues at the N-termini of the three target P450s were not included in model construction (see Additional files 1 and 2 for sequence alignment and percent sequence identity).

Comparative modeling of CYP6AA3, CYP6P7, and CYP6P8 was performed using a restrained-based approach implemented in MODELLER9v6 [13]. Multiple amino acid sequence alignment of CYP3A4, CYP2C8, and CYP2C9 template structures was performed using the SALIGN module in MODELLER9v6, and subsequently aligned individually with target enzymes. A set of 1000 models for each target enzyme was constructed. The coordinates of heme in the models were obtained from CYP3A4 (1TQN) and positioned in targets as in the 1TQN template. The resulting three-dimensional models of CYP6AA3, CYP6P7, and CYP6P8 were sorted according to scores calculated from discrete optimized protein energy (DOPE) scoring function [14]. The knowledge-based conditional probabilities for the residue specific all-atom probability discriminatory function (RAPDF) in RAMP suite was used to discriminate native structures from incorrectly-folded structures [15]. Refinement of models was performed using Amber10 package [16] to reduce steric clashes among residues. The AMBER ff03 all atom force field was applied. The proteins were solvated in TIP3P water molecules with 12 Å cutoff. Solvent was relaxed while backbone atoms were kept restrained for 100 steps of steepest decent followed by 200 steps of conjugated gradient. Subsequently, all atoms were allowed to move freely without any restraint until energy gradient was < 0.05 kcal/mol. The refined models were determined for distribution of phi and psi angles using ProSAII [17,18] and Procheck [19].

Three-dimensional structures of pyrethroids (permethrin, bioallethrin, cypermethrin, deltamethrin, and λ-cyhalothrin), alongside organophosphate (chlorpyrifos), and carbamate (propoxur) shown in Additional file 3 were obtained from ChemIDplus database http://chem.sis.nlm.nih.gov/chemidplus/ and used in docking of three target models. Partial charges of ligands and proteins were generated using Gasteiger method with the aid of AutoDockTools [20]. Restrained electrostatic potential atomic charge method described by Oda et al. [21] was used to assign high-spin state of five-coordinated ferrous heme complex to simulate substrate binding state. In addition, oxyferryl state was assigned to heme group of target models following Seifert et al. [22] to compare modes of substrate binding at different heme states. A cubic grid having 60 × 60 × 60 grid points per side and spacing of 0.375 Å was set corresponding to substrate recognition sites (SRSs) of each mosquito P450 model following those of CYP2 family proposed by Gotoh [23]. The second grid was positioned onto substrate access channels extending into binding pocket of individual model. Affinity maps of grids were calculated using AutoGrid program. AutoDock 4.0 program [24] was employed to dock ligands into active-site cavity of target models using Lamarckian genetic algorithm, consisting of 200 runs and 270000 generations, with the maximum number of energy evaluation set to 2.5 × 10^6^. Resulting docked conformations within 2.0 Å root mean square deviation (RMSD) tolerance were clustered and analyzed using AutoDockTools. Conformations with the lowest interaction energy and closest interaction to heme iron were selected. Residues showing interaction with docked ligands with less than 1.0 scaling factor of van der Waal radii were determined. Active sites and substrate access channels of enzyme models were calculated using the VOIDOO program [25] with conventional probe radius of 1.4 Å. Molecular visualization was performed on PyMOL 0.93 (Schrödinger, LLC).

## Results and Discussion

Homology models of CYP6AA3, CYP6P7 and CYP6P8 were constructed based on crystal structures of CYP3A4, CYP2C8, and CYP2C9 human P450s that are involved in pyrethroid metabolism [26] using the multiple sequence alignment strategy. Candidate predicted models of three mosquito P450s were selected based on the consensus judgment of DOPE and RAPDF scores that discriminate native structures from those misfolded. CYP6AA3, CYP6P7, and CYP6P8 models have overall ProSA z-scores of -8.24, -7.39, and -8.09, respectively. Ramachandran plot analyses of CYP6AA3, CYP6P7 and CYP6P8 models reveal 1.6%, 1.2% and 0.5% of the residues, respectively, located in disallowed regions. ProSA z-scores and Ramachandran plot analyses indicate that the three models are all of reasonable quality.

**Figure 1:**
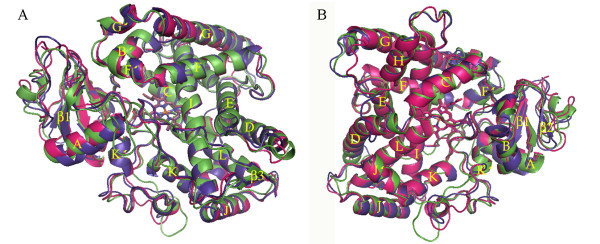
**Overall fold and overlay of homology models of CYP6AA3 (green), CYP6P7 (purple), and CYP6P8 (magenta)**. Model structures are shown in top (A) and back (B) views. Secondary structures of helices A-L and sheets β1-4 are labeled. The heme group in the middle of the structure is represented by stick.

**Figure 2:**
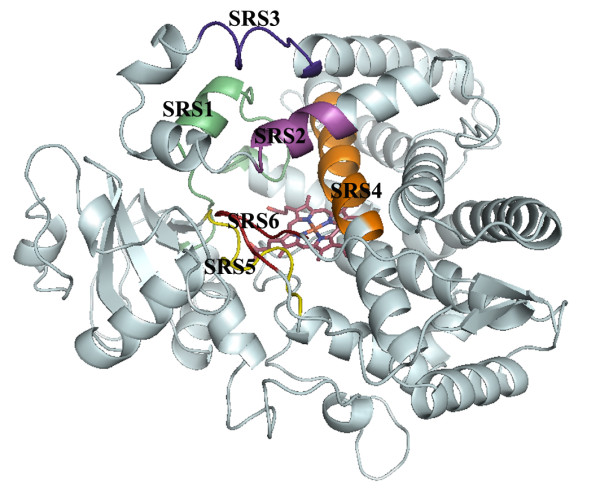
**Predicted substrate recognition sites (SRSs)**. SRSs are colored and designated 1-6 on homology model of CYP6AA3.

**Figure 3:**
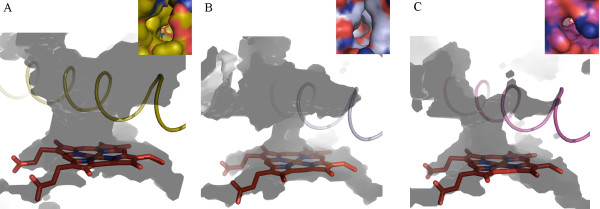
**Predicted active sites extending to enzyme surface of CYP6AA3 (A), CYP6P7 (B), and CYP6P8 (C)**. Active sites were calculated using VOIDOO. I-helices of CYP6AA3 (gold), CYP6P7 (silver), and CYP6P8 (magenta) are depicted in cartoon, spanning across heme. Caption in each figure corresponds to molecular surface view of each access channel. CYP6AA3 and CYP6P7 both display an oval shape of access opening, while CYP6P8 has a relatively small circular pore. The R114 guanidino group is shown projected horizontally to the opening of the CYP6P8 channel. Carbon atoms in caption are colored separately on each structure, oxygen and nitrogen atoms are shown in red and blue, respectively. The heme group in the middle of the structure is represented by red stick.

Overall conserved P450 folds are found in the three P450 models (Figure 1), such as helices D, E, I, J, K and L, and cysteine-pocket attaching heme. Structural differences between human and mosquito P450s are attributed to SRSs (Figure 2) spanning access channels and active sites of enzymes. Among the mosquito models, differences in substrate access channels and geometry of predicted active sites are apparent. Searching for possible substrate access channels revealed a surface channel opening in CYP6AA3, designated pw2c following a previous report [27], located between B^/^/C loop, C-terminus of G-helix and N-terminus of I-helix. The putative CYP6P7 pw2b access channel is comprised of residues from B/B^/^loop and β1 sheet, while pw2e is observed in BC loop/B^/^helix and N-terminus of I-helix of CYP6P8 model. Differences in the geometry of the predicted active sites is remarkable with CYP6AA3 having an oval shape and a large volume, 245.69 Å^3 ^in size (Figure 3A), while CYP6P7 has a restrained narrow opening to the heme prosthetic group and is smaller (volume of 135.11 Å^3^, Figure 3B). The CYP6P8 active site has the smallest volume, 68.13 Å^3^, attributed to protrusion of guanidino group of R216, and R114 is perpendicular to the channel opening (Figure 3C).

**Figure 4:**
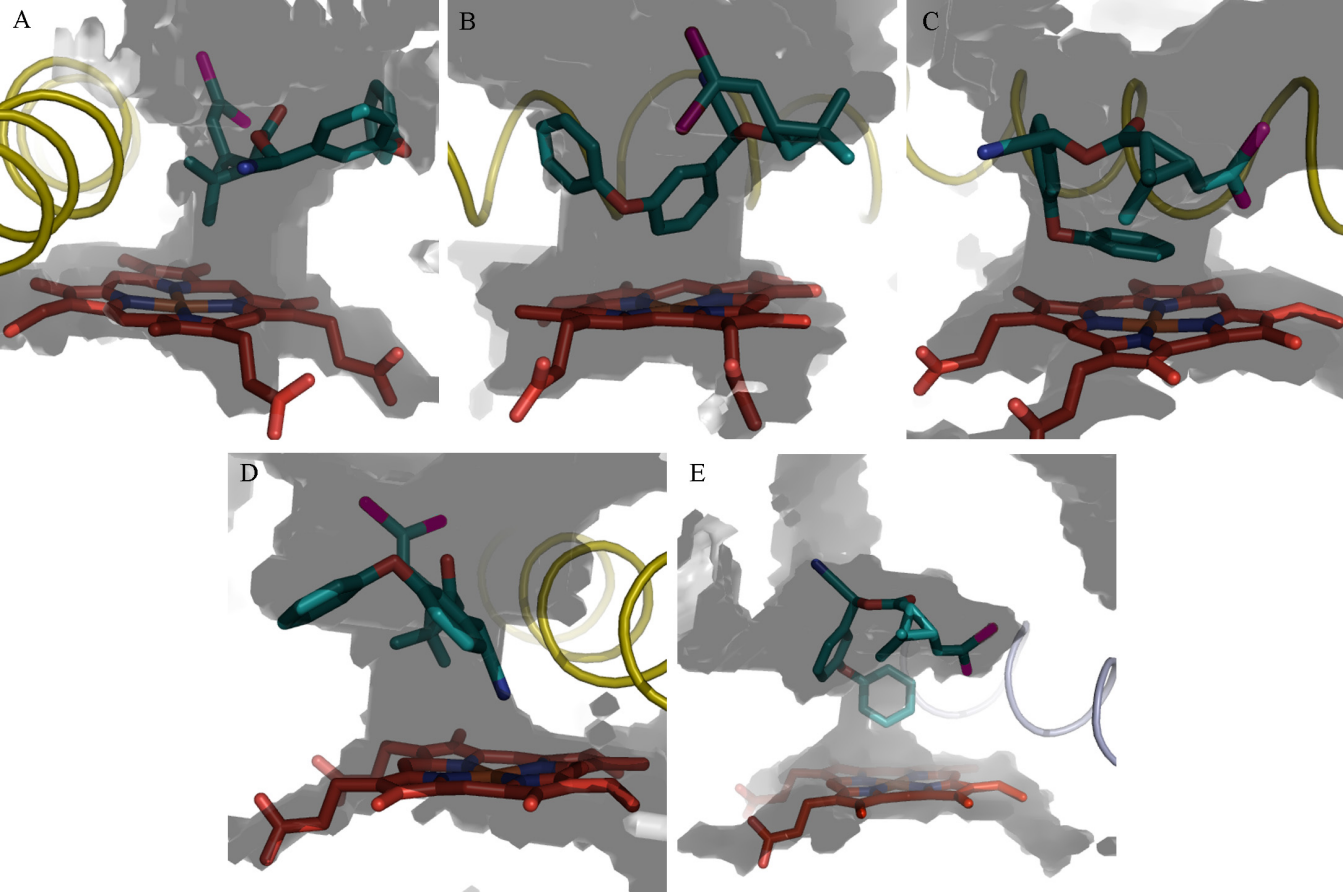
**Deltamethrin binding modes in active sites of CYP6AA3 (A-D) and CYP6P7 (E)**. CYP6AA3 exhibited 4 binding modes of deltamethrin positioning close to heme iron: geminal-dimethyl group (A), 5-phenoxybenzyl carbon (B), 4^/^-phenoxybenzyl carbon (C), and cyano group (D). Single binding mode of deltamethrin in CYP6P7 was obtained with 4^/^-phenoxybenzyl carbon as a predicted hydroxylation site (E). Same color is applied for heme and all elements as in Figure 3.

We have reported that CYP6AA3 and CYP6P7 can metabolize pyrethroids (permethrin, cypermethrin, and deltamethrin), but lacked activity against bioallethrin (pyrethroid), chlorpyrifos (organophosphate), and propoxur (carbamate) [5]. Notably CYP6AA3 contains λ-cyhalothrin (pyrethroid) degradation activity while CYP6P7 cannot metabolize this compound [5]. To compare the model structures of CYP6AA3, CYP6P7, and CYP6P8 with regard to their metabolic activities, pyrethroids were docked onto these models. In CYP6AA3, the large substrate channel allows passage of multiple conformations of pyrethroids to fit in active-site cavity, while CYP6P7 provides more restricted access of pyrethroids allowing one or two conformations to fit within the cavity (Table 1). Figure 4 shows an example of the predicted binding of deltamethrin, with multiple conformations, to the CYP6AA3 active site (Figure 4A-D) and a single deltamethrin conformation bound to CYP6P7 (Figure 4E). As such, it can be anticipated that CYP6AA3-mediated pyrethroid degradation has the potential to generate multiple metabolites according to the position of pyrethroids (geminal dimethyl group, 5- and 4^/^-phenoxybenzyl carbons, and alpha carbon at cyano group) attacked by the enzyme. This prediction is in agreement with our results showing multiple products from CYP6AA3-mediated deltamethrin degradation [28]. Multiple products have also been detected from pyrethroid metabolism mediated by insect and rodent P450s [4,29-31]. To test whether the binding of insecticides would be altered in different heme states of P450s, the oxyferryl state was simulated in CYP6AA3 and CYP6P7 and docked with deltamethrin. Our results indicate that the docked pyrethroid conformations on the iron-oxo enzyme complex (Additional file 4) are similar to those obtained from high-spin ferrous state (Figure 4), emphasizing the significance of enzyme active-site geometry to influence substrate selectivity and the conformation of substrate binding regardless of the heme state in the enzymes.

**Table 1:** Docking results of CYP6AA3 and CYP6P7 homology models

**Enzymes and insecticides**^**a**^	**Binding sites**^**b**^	Estimate free energy (kcal/mol)	**Distance from heme iron (Å)**^**c**^	**Predicted contact residues**^**d**^
CYP6AA3				
Permethrin	Gem	-7.31	3.26	(P217)^2^, R220, (F309, A310, T314)^4^, T317, (P375, V376)^5^, (M491)^6^
	C5-PB	-8.18	3.03	(H120)^1^, (V306, F309, A310, E313, T314)^4^, (P375)^5^, (L492)^6^
	C4^/^-PB	-7.86	3.97	(E112, P116, H120, F122)^1^, (F305, F309, A310, T314)^4^, (V376, I380)^5^
Cypermethrin	Gem	-9.10	3.01	(E112, P116, H120)^1^, (R220)^2^, (F309, A310, T314)^4^, (I380, R381)^5^
	C5-PB	-9.6	3.14	(F122)^1^, (A310, T314)^4^, (P375, V376, P377, R381)^5^, (M491, L492)^6^
	C4^/^-PB	-9.64	4.38	(E112, H120, F122)^1^, (V306, F309, A310, T314)^4^, (V376, I380, R381, V382)^5^
	CN	-9.41	4.09	(H120, F122)^1^, (V306, F309, A310, T314)^4^, (P375, V376)^5^, (L492)^6^
Deltamethrin	Gem	-8.50	3.45	(E112, H120, F122)^1^, (A310, E313, T314)^4^, (P375, V376, I380, R381)^5^, (M491)^6^
	C5-PB	-8.66	3.31	(H120, F122)^1^, (F309, A310, T314)^4^, (V376, Q378, I380, R381)^5^
	C4^/^-PB	-8.52	3.49	(F122)^1^, (A310, T314)^4^, (P375, V376, I380, R381, V382)^5^, (M491, L492)^6^
	CN	-8.74	4.35	(H120, F122)^1^, (F309, A310, T314)^4^, (P375, V376, P377, Q378, I380, R381)^5^
λ-cyhalothrin	Gem	-7.90	3.31	(H120, F122)^1^, (P217, N221)^2^, (F309, A310, T314)^4^, (P375, V376)^5^, (M491, L492)^6^
	C5-PB	-6.94	3.82	(H120, F122)^1^, (T314)^4^, (V376, R381)^5^
	C4^/^-PB	-7.65	3.02	(Y109, E112)^1^, (R220)^2^, (F309, A310, T314)^4^, (P375, V376, I380, R381)^5^
	CN	-7.89	3.51	(H120, F122)^1^, (V306, F309, A310, E313, T314)^4^, T317, (P375, V376, I380)^5^, (L492)^6^
CYP6P7				
Permethrin	C5-PB	-6.45	3.35	(L313, A314, E317, T318)^4^, (L380, E381, S382, I383, R385)^5^, (F494, I495)^6^
Cypermethrin	Gem	-8.92	3.52	(F110, F123)^1^, (T220)^2^, (L313, A314, T318)^4^, (E381, R385)^5^, (F494)^6^
	C4^/^-PB	-9.50	3.31	(L313, A314, E317, T318)^4^, (L380, E381, S382, I383, R385)^5^, (F494, I495)^6^
Deltamethrin	C4^/^-PB	-8.17	3.53	(F123)^1^, (T220)^2^, (A314, T318)^4^, (L380, E381, R385)^5^, (F494, I495, L496)^6^
λ-cyhalothrin	C4^/^-PB	-8.52	4.17	(E317, T318)^4^, T321, (L380, E381, S382, R385)^5^, (I495, L496)^6^

^a^Chemical structures of insecticides are shown in Additional file 3.

^b^Predicted metabolic sites are indicated as following: Gem, geminal-dimethyl group in acid moiety; C5-PB, carbon 5 of phenoxybenzyl group in alcohol moiety; C4^/^-PB, carbon 4^/^of phenoxybenzyl group in alcohol moiety; and CN, cyano group. ^c^Distances between the heme iron and putative metabolic sites are measured.

^d^Residues are grouped based on substrate recognition sites (SRSs) in parentheses. Superscript represents order of SRS.

Compared to CYP6AA3, CYP6P7 possesses a narrow channel opening to heme iron, resulting in structural constraint toward pyrethroids and limited access to λ-cyhalothrin. The phenoxybenzyl moiety of pyrethroids is predicted to be a favorable attack site for CYP6P7 (Table 1). Moreover the bulky trifluoromethyl group (Additional file 3) causes the 4^/^-phenoxybenzyl carbon of λ-cyhalothrin moving away from the CYP6P7 heme (4.17 Å) compared to that of cypermethrin (3.31 Å, Table 1), and thus the trifluoromethyl group may be responsible for absence of detectable CYP6P7 activity against λ-cyhalothrin [5]. Analogous findings have been shown for a CYP6B8v1 predicted model that contains a narrow active-site cavity, leading to its ability to metabolize only small flexible molecules but not large rigid molecules [7].

Residues in the CYP6AA3 cavity that interact with pyrethroids in our docking experiments (Table 1) are generally non-polar, implying that binding may occur via hydrophobic interactions with non-polar pyrethroids that have octanol-water partition coefficient (log *P*) values ranging from 6.2-6.8. Since bioallethrin, chlorpyrifos, and propoxur (values of 4.78, 4.96, and 1.52 respectively) are more polar, they would be expected to have less favorable interactions with the active sites of CYP6AA3 and CYP6P7 resulting in a longer distance between these molecules and the heme iron (unpublished data). As a result both CYP6AA3 and CYP6P7 are predicted to be incapable of metabolizing bioallethrin, chlorpyrifos, and propoxur, consistent with our previous results [5]. Binding of thiodicarb (carbamate, log *P *of 1.62), temephos, and fenitrothion (organophosphates, log *P *of 5.96 and 3.3, respectively) to both of these enzymes is also predicted to be unfavorable (unpublished data).

**Figure 5:**
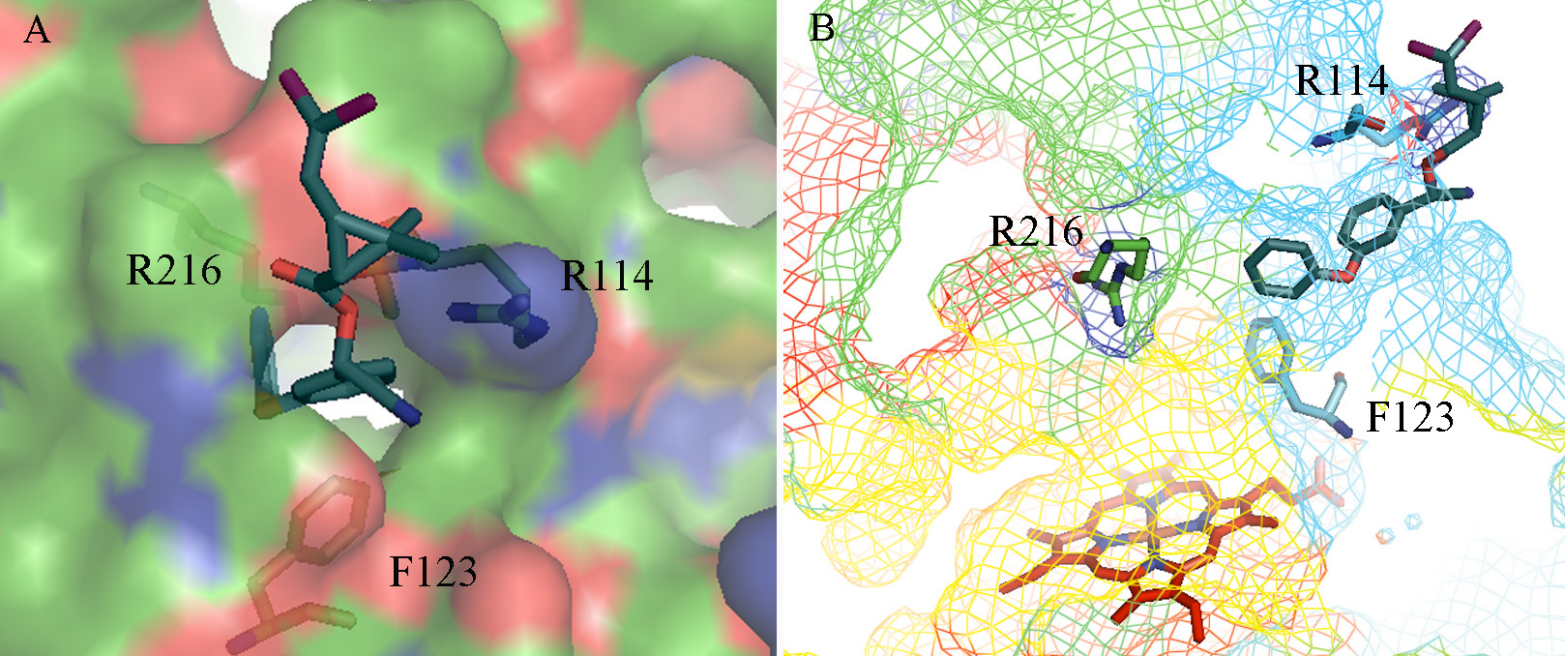
**Obstruction of deltamethrin entry into CYP6P8 active site**. (A) Predicted hydrogen bond formation between oxygen on ester of deltamethrin and guanidino group of R114. (B) Presence of R216 located across F123 in narrow active site channel. CYP6P8 is in rainbow mesh, ranging from blue (N-terminal) to red (C-terminal). Deltamethrin is represented by teal stick.

In the CYP6P8 model, the small access channel together with R114 and R216 are predicted to obstruct pyrethroid entry into the active-site cavity (Figure 5), resulting in the absence of CYP6P8 activity toward pyrethroid [5]. Equivalent R114 and R216 residues are not found in CYP6AA3 and CYP6P7 active sites and no such hindrance of pyrethroid access is predicted. The presence of the positively charged guanidino group of R114, speculated to form hydrogen bond with the oxygen on ester of pyrethroid, and R216 lying in narrow path (6.75 Å-width across F123) are predicted to impede pyrethroids from passing through the CYP6P8 channel opening (Figure 5B). A similar situation has been observed in protein kinase C, where mutation of amino acids to arginine residues in the C1 domain binding cleft can significantly reduce interaction and membrane translocation of phorbol 12,13-dibutyrate [32]. It is possible that the topology and residues within the CYP6P8 active site might not favorably allow entry of arene compounds such as pyrethroids to the heme center, but may allow entry of small hydrocarbon compounds. Further investigation of CYP6P8-mediated metabolism may reveal its preferred substrates.

## Conclusion

The model structures of CYP6AA3, CYP6P7, and CYP6P8 generated in this study have allowed us to better understand the different substrate preferences between these P450 enzymes and the predictions based on our docking studies are consistent with experimental results of pyrethroid metabolism mediated by these three enzymes. Variations in the predicted substrate channels and geometry of active sites appear to be responsible for their differences in binding to pyrethroids. Our findings indicate that differences in metabolic activities among P450 enzymes in insects can be attributed to structural differences that allows for selectivity in their activities against insecticides. These models have the potential to be used in the investigation of candidate P450 inhibitors or in the analysis of the binding and metabolism of insecticide compounds that have potential for use in the control of the mosquito vector.

## Competing interests

The authors declare that they have no competing interests.

## Authors' contributions

PL participated in design of the study, model building and data analysis, and drafting of the manuscript. EJ participated in design and data analysis of the study. PR participated in design and data analysis of the study, drafting and revising of the manuscript. All authors read and approved the final manuscript.

## Supplementary Material

Additional file 1 **Figure S1**. Multiple sequence alignment of templates and target P450s. Template sequences of CYP3A4 (1TQN chain A), CYP2C9 (1OG2 chain A) and CYP2C8 (1PQN chain A) are aligned against target sequences CYP6AA3, CYP6P7 and CYP6P8. The first 25 residues on N-termini of target sequences are underlined and bolded. Residues mostly identical among templates and targets are marked yellow. Identical residues among all sequences are indicated by asterisks and yellow mark. Residues of target identical to CYP3A4 template are marked grey, whereas target residues identical to CYP2Cs are marked green. Predicted contact residues between targets and ligands are marked blue. Protruding arginine at the entry of substrate access channel in CYP6P8 model is highlighted red and arginine and phenylalanine in the channel are in violet. CYP6AA3 primary sequence comprises 505 residues, CYP6P7 509 residues and CYP6P8 506 residues. Click here for file

Additional file 2 **Table**. Percent amino acid sequence similarity between crystallographic templates and target sequences. Click here for file

Additional file 3 **Figure S2**. Insecticides used in docking study. Pyrethroids shown are cypermethrin (A), permethrin (B), deltamethrin (C) and λ-cyhalothrin (D). Non-substrate insecticides docked in this study are: propoxur, a type of carbamate insecticide (E); chlorpyrifos, a type of organophosphate insecticide (F); and bioallethrin pyrethroid insecticide (G). Geminal-dimethyl group, 5- and 4^/^-phenoxybenzyl carbons are indicated by arrows on cypermethrin. Click here for file

Additional file 4 **Figure S3**. Docked deltamethrin conformations in oxyferryl state of CYP6AA3 (A-D) and CYP6P7 (E). Predicted metabolic sites are at geminal dimethyl group (A), 5-phenoxybenzyl carbon (B), 4^/^-phenoxybenzyl carbon (C), cyano group (D), and 4^/^-phenoxybenzyl carbon in CYP6P7 (E). Helices I of CYP6AA3 and CYP6P7 are shown in gold and silver cartoons, respectively. Oxyferryl heme is represented by grey stick, while deltamethrin is illustrated in green stick. Iron, oxygen, and nitrogen are colored orange, red, and blue, respectively. Click here for file
