# Supplementary figures and images for: Homology modeling of mosquito cytochrome P450 enzymes involved in pyrethroid metabolism: insights into differences in substrate selectivity
Source: BMC Res Notes. 2011 Sep 6;4:321. doi: 10.1186/1756-0500-4-321 (PMC3228512; doi:10.1186/1756-0500-4-321)

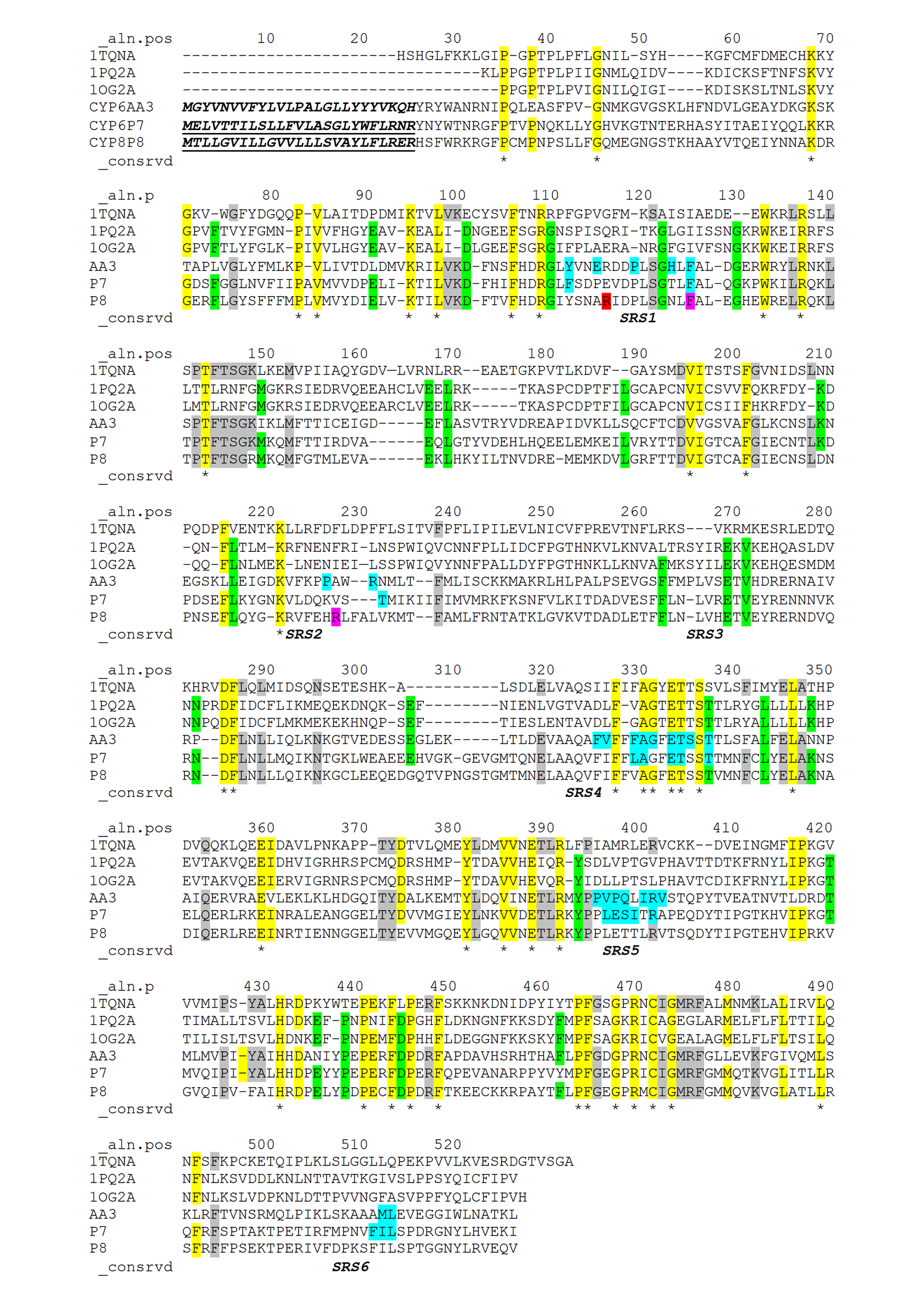

Supplement: Additional file 1 — Figure S1. Multiple sequence alignment of templates and target P450s. Template sequences of CYP3A4 (1TQN chain A), CYP2C9 (1OG2 chain A) and CYP2C8 (1PQN chain A) are aligned against target sequences CYP6AA3, CYP6P7 and CYP6P8. The first 25 residues on N-termini of target sequences are underlined and bolded. Residues mostly identical among templates and targets are marked yellow. Identical residues among all sequences are indicated by asterisks and yellow mark. Residues of target identical to CYP3A4 template are marked grey, whereas target residues identical to CYP2Cs are marked green. Predicted contact residues between targets and ligands are marked blue. Protruding arginine at the entry of substrate access channel in CYP6P8 model is highlighted red and arginine and phenylalanine in the channel are in violet. CYP6AA3 primary sequence comprises 505 residues, CYP6P7 509 residues and CYP6P8 506 residues. [file 1756-0500-4-321-S1.TIFF]

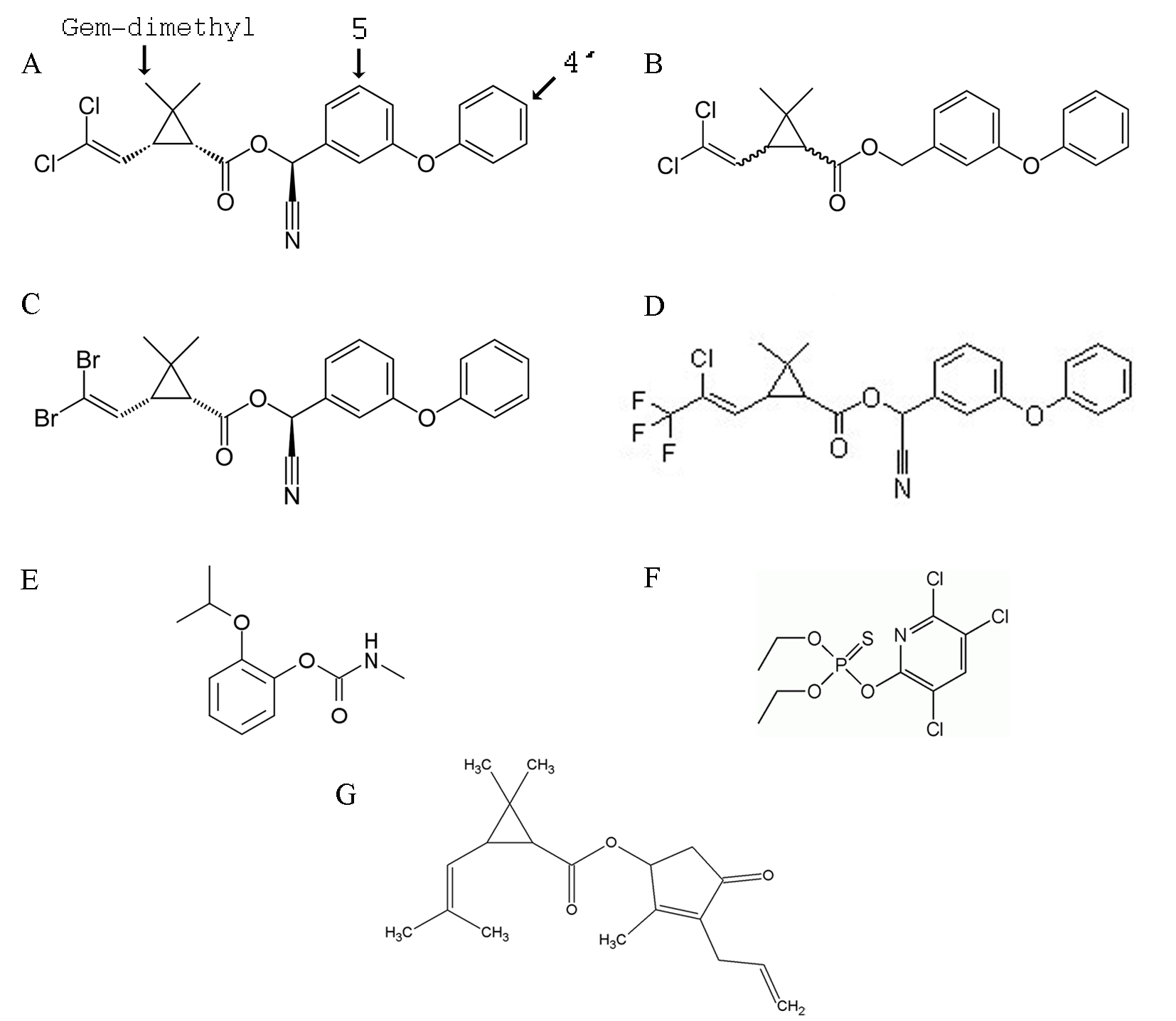

Supplement: Additional file 3 — Figure S2. Insecticides used in docking study. Pyrethroids shown are cypermethrin (A), permethrin (B), deltamethrin (C) and λ-cyhalothrin (D). Non-substrate insecticides docked in this study are: propoxur, a type of carbamate insecticide (E); chlorpyrifos, a type of organophosphate insecticide (F); and bioallethrin pyrethroid insecticide (G). Geminal-dimethyl group, 5- and 4/-phenoxybenzyl carbons are indicated by arrows on cypermethrin. [file 1756-0500-4-321-S3.TIFF]

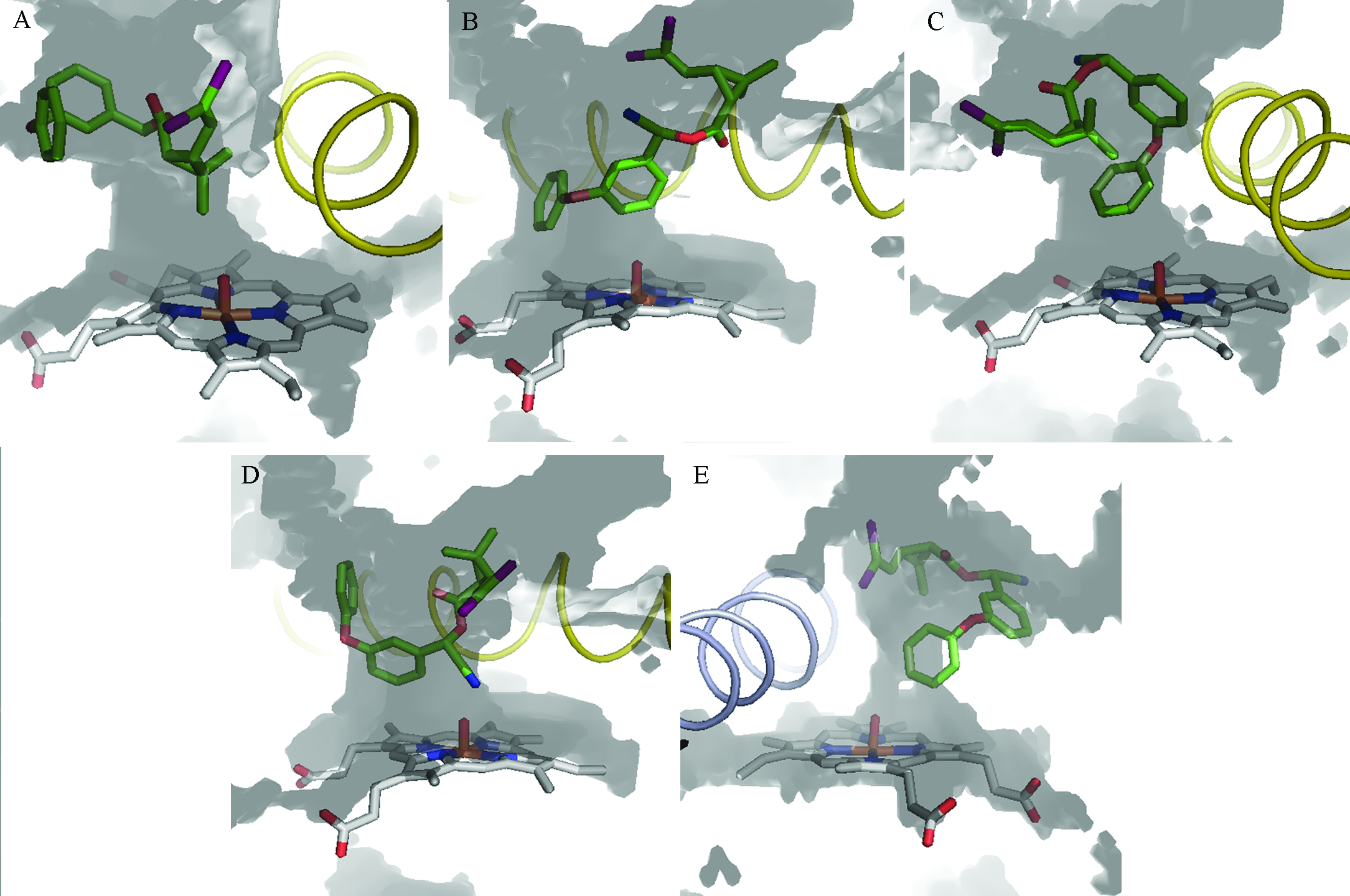

Supplement: Additional file 4 — Figure S3. Docked deltamethrin conformations in oxyferryl state of CYP6AA3 (A-D) and CYP6P7 (E). Predicted metabolic sites are at geminal dimethyl group (A), 5-phenoxybenzyl carbon (B), 4/-phenoxybenzyl carbon (C), cyano group (D), and 4/-phenoxybenzyl carbon in CYP6P7 (E). Helices I of CYP6AA3 and CYP6P7 are shown in gold and silver cartoons, respectively. Oxyferryl heme is represented by grey stick, while deltamethrin is illustrated in green stick. Iron, oxygen, and nitrogen are colored orange, red, and blue, respectively. [file 1756-0500-4-321-S4.TIFF]
