# Supplementary material for: Homology modeling of mosquito cytochrome P450 enzymes involved in pyrethroid metabolism: insights into differences in substrate selectivity
Source: BMC Res Notes. 2011 Sep 6;4:321. doi: 10.1186/1756-0500-4-321 (PMC3228512; doi:10.1186/1756-0500-4-321)
Supplement: Additional file 2 — Table. Percent amino acid sequence similarity between crystallographic templates and target sequences. [file 1756-0500-4-321-S2.DOC]

**Percent amino acid sequence similarity between crystallographic templates and target sequences**

| **Target sequence** | **CYP3A4** | **CYP2C8** | **CYP2C9** | **CYP6P7** | **CYP6P8** |
| --- | --- | --- | --- | --- | --- |
| CYP6AA3 | 33 | 22 | 22 | 40 % | 41% |
| CYP6P7 | 22 | 16 | 16 | - | 61% |
| CYP6P8 | 20 | 14 | 16 | 61% | - |
